# Supplementary material for: The causal role of circulating inflammatory markers in osteoporosis: a bidirectional Mendelian randomized study
Source: Front Immunol. 2024 Jul 18;15:1412298. doi: 10.3389/fimmu.2024.1412298 (PMC11291241; doi:10.3389/fimmu.2024.1412298)
Supplement: Supplementary file 1 [file DataSheet_1.zip › supplementary material/OSTEOPOROSIS-CIM/GCST90274838.tsv.gz_forest.pdf]

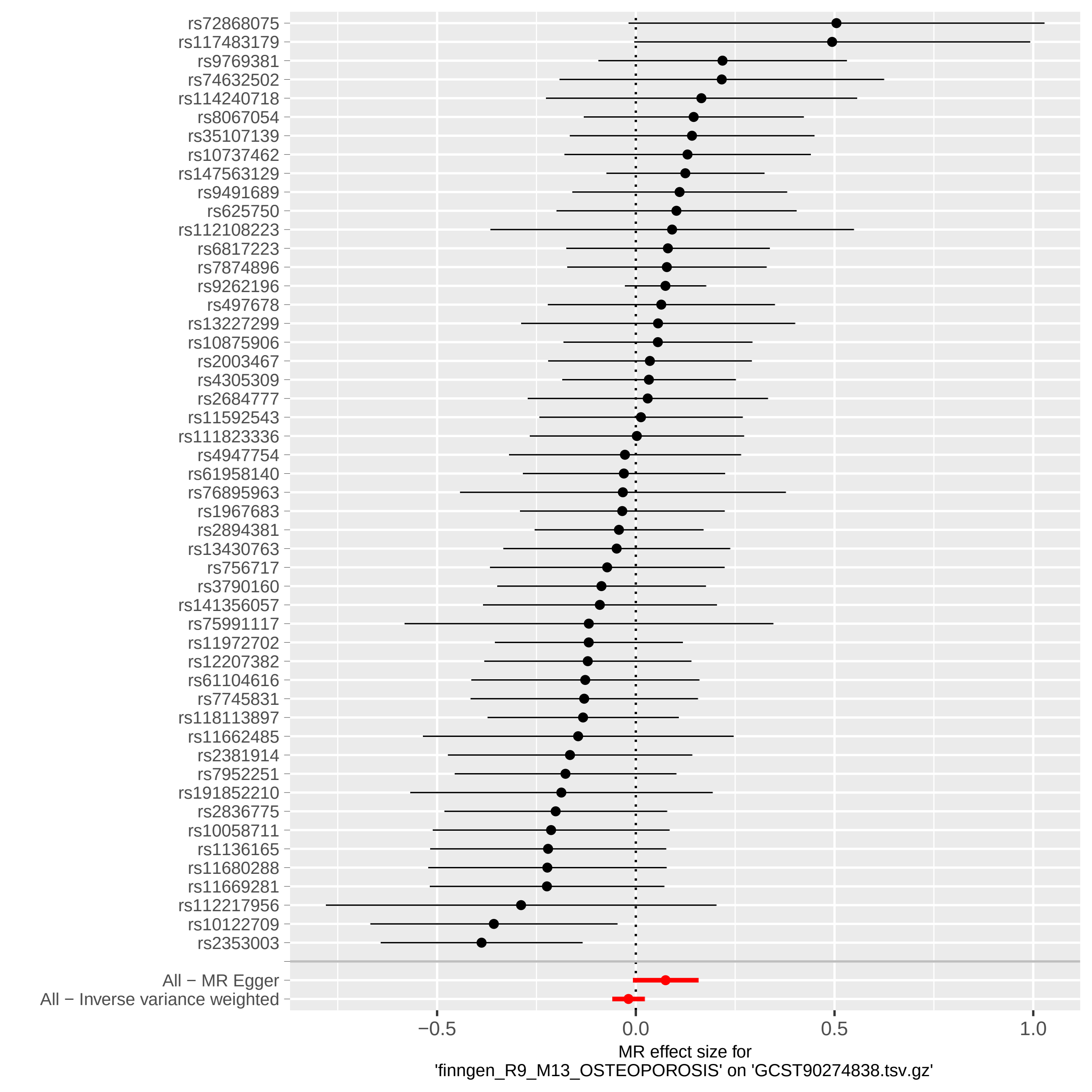
rs72868075
rs117483179
rs9769381
rs74632502
rs114240718
rs8067054
rs35107139
rs10737462
rs147563129
rs9491689
rs625750
rs112108223
rs6817223
rs7874896
rs9262196
rs497678
rs13227299
rs10875906
rs2003467
rs4305309
rs2684777
rs11592543
rs111823336
rs4947754
rs61958140
rs76895963
rs1967683
rs2894381
rs13430763
rs756717
rs3790160
rs141356057
rs75991117
rs11972702
rs12207382
rs61104616
rs7745831
rs118113897
rs11662485
rs2381914
rs7952251
rs191852210
rs2836775
rs10058711
rs1136165
rs11680288
rs11669281
rs112217956
rs10122709
rs2353003
All − MR Egger
All − Inverse variance weighted
−0.5
0.0
0.5
1.0
MR effect size for
'finngen_R9_M13_OSTEOPOROSIS' on 'GCST90274838.tsv.gz'
